# Biological Monitoring of Blood Naphthalene Levels as a Marker of Occupational Exposure to PAHs among Auto-Mechanics and Spray Painters in Rawalpindi

**DOI:** 10.1186/1471-2458-11-467

**Published:** 2011-06-13

**Authors:** Atif Kamal, Mazhar Qayyum, Iqbal U Cheema, Audil Rashid

**Affiliations:** 1 Department of Environmental Sciences, Pir Mehr Ali Shah Arid Agriculture University, Rawalpindi, Pakistan; 2 Department of Biology, Pir Mehr Ali Shah Arid Agriculture University, Rawalpindi, Pakistan

## Abstract

**Background:**

Routine exposure to chemical contaminants in workplace is a cause for concern over potential health risks to workers. In Pakistan, reports on occupational exposure and related health risks are almost non-existent, which reflects the scarce availability of survey data and criteria for determining whether an unsafe exposure has occurred. The current study was designed to evaluate blood naphthalene (NAPH) levels as an indicator of exposure to polycyclic aromatic hydrocarbons (PAHs) among automobile workshop mechanics (MCs) and car-spray painters (PNs). We further determined the relationship between blood NAPH levels and personal behavioural, job related parameters and various environmental factors that may further be associated with elevated risks of occupational exposures to PAHs.

**Methods:**

Sixty blood samples (n = 20 for each group i.e. MC, PN and control group) were collected to compare their blood NAPH levels among exposed (MCs and PNs) and un-exposed (control) groups. Samples were analyzed using high pressure liquid chromatography (HPLC). Data regarding demographic aspects of the subjects and their socioeconomic features were collected using a questionnaire. Subjects were also asked to report environmental hygiene conditions of their occupational environment.

**Results:**

We identified automobile work areas as potential sites for PAHs exposure, which was reflected by higher blood NAPH levels among MCs. Blood NAPH levels ranged from 53.7 to 1980.6 μgL^-1 ^and 54.1 to 892.9 μgL^-1 ^among MCs and PNs respectively. Comparison within each group showed that smoking enhanced exposure risks several fold and both active and passive smoking were among personal parameters that were significantly correlated with log-transformed blood NAPH levels. For exposed groups, work hours and work experience were job related parameters that showed strong associations with the increase in blood NAPH levels. Poor workplace hygiene and ventilation were recognized as most significant predictors related to differences among workplaces that may enhance the extent of exposure to chemical contaminants.

**Conclusions:**

It appeared that chemical exposure at the workplace may be influenced by multiple environmental factors, but poor workplace hygiene and duration of exposure (long work hours) were the most important factors. Smoking and negligence of workers regarding self protection were among some of the important personal behaviours than can be addressed with better training. There is also a need to improve workplaces hygiene and to rationalize work hours to minimize health risks. Since smoking was an important confounding factor that supplemented most of the actual occupational exposure, a study based on non-smoker subjects is needed to separate out the effects of smoking and other confounding factors that may obscure measurements of actual extent of occupational exposure.

## Background

Occupational exposures to hazardous chemicals are common in industries using solvent based materials as well as in indoor environments where people are exposed to volatile organic compounds from various sources [1]. Among the most common contaminants PAHs are of particular concern as they are comprised of a group of highly lipophilic, non-polar and persistent substances with remarkable mutagenic and carcinogenic properties. Exposure of PAHs can come from both occupational and environmental sources arising from use of coal tar, fires at oil wells, exposure to automotive exhaust gases, tobacco smoke and crude and mineral oils as well as incomplete combustion of fossil fuel and many other sources [2,3]. Among the attributes of PAHs that determine the bioavailability and toxicity of individual chemicals, molecular weight is the most important, as most of the higher molecular weight PAHs usually settled with particulate matter, and those with low molecular weight remain suspended in the air where they can become disseminated in the environment and inhaled by human [4,5]. Among low molecular weight PAHs, especially NAPH is the major PAH in motor exhaust gas [6]. NAPH being a potential carcinogen [7] is easily dispersed in gaseous phase and is abundantly found at industrial sites [8]. Studies have shown that NAPH poisoning in humans may lead to health risks such as haemolytic anaemia, nephrotoxicity, ophthalmologic and dermal changes [9]. Moreover, other environmentally widespread members of PAHs also pose a negative impact on the human immune system [10].

Although the rate of occupational diseases and injuries is very high in Pakistan, and thousands of workers are routinely exposed to various chemicals, there is currently little or no survey data due to lack of proper reporting or monitoring procedures [11]. Nonetheless, it is important to create better awareness of occupational hazards among workers to promote occupational safety [12]. Since no published data is available on biological monitoring of exposure to PAHs in Pakistan, we consider it to be one of the most neglected aspects in our occupational settings. Despite efforts, we could not find any proper set of rules and regulations for worker safety in any of our visited workplaces. Lack of awareness regarding chemical hazards among workers is a general consequence of such weaknesses in occupational surveillance system. In the this study, a survey of selected workplaces i.e. car-mechanics (MCs) and car-spray painters (PNs) workshops was conducted to gain general information on chemicals and workplace prevailing conditions that may be potential hazards to these workers. In addition to this, biological monitoring of blood NAPH levels was performed to estimate the extent of personal exposure to chemicals in the workplace environment. An effort was also made to identify an association between blood NAPH levels and predictor variables (socio-economic and environmental) that may be helpful in determining cause and effect of occupational hazards. In the light of the research outcome, this study further focused on highlighting priorities that could be helpful to ensure better safety to the workers in their workplaces and to minimize work-related health risks.

## Methods

### Questionnaire design and data collection

A brief questionnaire was designed to facilitate interviews of each worker to obtain information on their socio-demographic status and workplace environmental hygiene conditions. The workplace hygiene was monitored during sampling and was ranked as 'good, 'satisfactory' or 'poor'. All the workplaces were visited and monitored by the same investigator (AK) during sampling and questionnaire data acquisition. Hygiene conditions were ranked by following pre-set criteria assigned to workplace floors, ceiling walls and overall contamination at work areas. The floors heavily contaminated with chemical residue and other debris were given the lowest rank (i.e. poor). Middle and highest rakings were given to those areas which were kept chemical free to greater extent and were maintained properly i.e. separate working and storage areas with distinguished chemical disposal areas. Similar criteria were adopted for the workplace furniture, wall and ceiling rankings. Since visual observation is the simplest and common way to initiate the assessment of the expected hazards, we observed both the workers behaviour and the workplace conditions during performance of routine tasks [13]. Evaluation of work place criteria included assessment of general maintenance and chemical handling, evidence of spills or residues, presence of exhaust facilities and over all conditions of walls, ceiling and floor, and air circulation across work areas all of which were recorded through the questionnaire as numerical rankings. Worker safety was assessed by observing the use of self-protective equipment like gloves, masks, etc. as a routine safety protocol for personal protection. The questionnaires were signed by each subject to document their agreement with the assessment and consent. Blood sampling was done at the most separate and safe location of the work place (in most case in the office of supervisor) attending only one subject at a time. A standard medical kit and all precautionary measures (use of sterile syringes, vacutainer, disposable gloves, alcoholic swabs and safety bandages, etc.) were adopted during sample withdrawal to avoid any chance of contamination. Since blood was immediately transferred into vacutainer and stored in the iced box, there was little chance for contamination.

### Sampling

Sampling sites were divided into control and exposed areas. We collected sixty blood samples from three groups, i.e. MCs, PNs and non-exposed control group (n = 20 from each group). Selection of control subjects included workers from non-chemical occupations, such as retailers, book vendors, greengrocers, tailors, etc. who work indoors similar hours but in workplace conditions that are relatively free of chemicals. All samples were taken on the spot during regular work hours with the consent of subjects and willingness of their supervisors, after having clearly describing the purpose of study and expected outcomes. Blood samples (3-4 ml) were taken from the antecubital vein of each subject and were immediately transferred into vacutainer containing no anticoagulants. All these blood samples were transferred to the experimental laboratory as early as possible. Serum separation was carried out at 3000 rpm, after which it was frozen in 1.5 ml eppendorf tubes until the samples were further processed for analysis. None of the workers was found to be equipped with any type of self protective equipments.

### Chemicals

Chemicals used for the extraction of NAPH and sample cleanup from blood were HPLC or analytical grade i.e. n-hexane (Merck, No: 843, 95% pure), diethyl ether (Assay-GC 99.5% pure, Riedel-de Haen), sodium sulphate anhydrous (99% pure; Panreac) and methanol (Assay-GC 99.7% pure; Riedel-de Haen). Acetonitrile (MW 41.05 HPLC grade; Sigma Aldrich) was used for final reconstitution of samples and also as mobile phase with water (UV-HPLC PAI-ACS Panreac, M = 18.016). NAPH Standard (C_10_H_8,_, GDR, MW. 128.17, GLC purity 99.8%) was purchased from BDH Laboratory supplies (pool BH-15 ITD England). Stock was prepared in acetonitrile from which calibration standards were prepared by serial dilutions. Prepared standards (100 μg ml^-1^) were run a number of times (thrice a single day, for 3-4 days) to optimize signal to noise ratio (improved by limiting pumps pulsation, and mobile phase optimization). For the determination of limit of detection (LOD) and that of quantification (LOQ), signal to noise ratio (S/N) method was used. Briefly 5-6 blanks were run followed by running the same number of standard samples to get average height of baseline noise and the standard respectively. The quotient of standard and baseline-noise heights represented the LOD (= 0.02 μg ml^-1^) and LOQ (= 0.6 μg ml^-1^) at 3:1 and 10:1 ratios respectively. A few samples were also spiked with known standard concentration to determine percentage recovery (measured as 89-96%) and to assure accuracy and precision. Each run was initiated with a stabilized base line and a blank run prior to running the actual samples.

### Analytical procedures

Every possible effort was made to avoid contamination and to observe good laboratory practices. All of the extraction and clean up steps were standardized and checked for optimum behaviour. Samples were prepared according to the method reported by Al-Daghri (2008) and analyzed using HPLC (SPD-10A VP-Shimadzu) equipped with an RP-C18 column, auto-injector (Shimadzu SIL-10 VP) UV/VIS Detector (Shimadzu SPD-10AVP, at 254 nm) using acetonitrile as mobile phase under varying concentrations with water (UV-HPLC PAI-ACS Panreac, M = 18.016) at flow rate of 1 ml min^-1 ^in isocratic elution mode (P.I = 8.32) at ambient temperature. Blood NAPH in the samples was identified on the basis of respective retention times quantified on the basis of respective peak areas and expressed as μgL^-1^, using following formula [14].

### Statistical analysis

The difference between categorical variables related to socio-economic and demographic characteristics were calculated using chi-square test. Mann-Whitney U-test was used to compare the NAPH concentration among blood samples of each group. Spearman correlation test was performed to examine the relationships between blood contamination level and smoking habits, job duration etc. Data from exposed group were also log-transformed to improve normality. An alpha level of P < 0.05 was considered as statistically significant. For tabulation of the data, Microsoft Excel spreadsheets were used and statistical analyses were performed using SPSS Version-12.

## Ethics

Samples were taken only form subjects who were wiling to participate in this study. The purpose of investigations and expected outcomes were clearly explained prior to sampling. All subjects singed the questionnaire as their written consent. Permission and logistic support for this research was granted by the ethical review committee of the University in collaboration with Volunteer's Social Welfare Organization (VSWO) (registered under Social Welfare Act. 1961, Pakistan) http://thevolunteers.webs.com.

## Results

All participants in the selected occupations were males; therefore the control group was also comprised of male participants. In Table 1, the categorical variables from each exposed and un-exposed (control) are described. These variables initially were analyzed with respect to demographic and socio-economic differences using the χ^2^-test. A significantly lower level of education was observed among MCs and PNs in comparison to the control group (P < 0.001 and P < 0.001, respectively) with lowest education level in PNs. Comparisons for income status showed that PNs had poor income (P < 0.001), scarcely meeting out expenses with their monthly income. Most of the subjects in exposed groups did not have their own home, of which the greater percentage was observed in MCs (P < 0.05). The smoking habit was more prevalent among PNs as compared to control group (P < 0.005) while this difference was not statistically significant between MCs and control subjects (Table 1). Presence of any other smokers at home (i.e. being a passive smoker) was found to be higher in MCs group, whereas no difference was observed in age, height, weight and BMI among the participants. It was observed during sample collection that none of the workers from occupationally exposed groups (MCs and PNs) was equipped with any type of protective equipment.

**Table 1:** Personal characteristics of subjects in exposed groups (car-spray painters and car-mechanics) vs. control group

Parameters	Car-Spray painters (n = 20)	Car-Mechanics (n = 20)	Control (n = 20)	**P-value **^**a**^	**P-value **^**b**^
Age (years)	27.5 (25.3-29)*	28.10 (25.25-30.8)	26.85 (22.3-29.7)	0.51 ^c^	0.21 ^c^
Height (cm)	171 (165-176)	170.6 (165-173.7)	171 (164-176)	0.77 ^c^	1.00 ^c^
Weight in (Kg)	61 (56-62.75)	58.50 (55-62)	59.50 (55-65)	0.84 ^d^	0.67 ^d^
BMI (kg/m^2^)	18.9 (18.2-20.3)	19.05 (18.4-20.7)	19.10 (18.2-20.9)	0.88 ^c^	0.92 ^c^
Income (× 10^3^) Rs.	8.5 (6.5-11.5)	8.50 (8-10)	14 (12.3-16)	<0.001 ^e^	<0.001 ^e^
**Expenditure versus Income**					
Low	7 (35) **	2 (10)	4 (20)	<0.004 ^e^	<0.02 ^e^
Equal	4 (20)	8 (40)	14 (70)		
High	9 (45)	10 (50)	2 (10)		
**Education Level**					
Under-Primary	16 (80) **	8 (40)	0 (0)	<0.001 ^e^	<0.001 ^e^
Primary	3 (15)	7 (35)	3 (15)		
Secondary	1 (5)	4 (20)	12 (60)		
Higher-secondary	0 (0)	1 (5)	5 (25)		
**Home ownership**					
No	11 (55)	13 (65)	5 (25)	<0.05 ^e^	<0.01 ^e^
Yes	9 (45)	7 (35)	15 (75)		
**Smoking Status**					
Non-smoker	8 (40)**	11 (55)	17 (85)	<0.005 ^e^	0.225 ^e^
Smoker	12 (60)	9 (45)	3 (15)		
**Passive Smoking (at home)**					
No	12 (60)	10 (50)	16 (80)	<0.150 ^e^	<0.048 ^e^
Yes	8 (40)	10 (50)	4 (20)	*r = 0. 59 *^f^	*r = 0.62 *^f^

* Median (25th-75th)

** Frequency (% of total)

^a ^Car-spray painters v/s Control P-values

^b ^Car-mechanics v/s Control, P-values

^c ^Wilcoxon Signed Rank Test

^d ^Welch test

^e ^Chi-square test

^f ^= correlation between blood NAPH and presence of smoker at home

None of the work locations could be classified into the good hygiene category. Our survey of various workplaces showed that among all the areas visited only ten percent were satisfactory regarding prevailing hygiene, whereas ninety percent provided poor hygiene conditions (Table 2). Workplace hygiene among control and exposed areas was also poor (P < 0.001 painting areas; P < 0.001 in MCs workshops) as compared to un-exposed (control) areas (Table 2). Ventilation status in the exposed workplaces were generally poor and were also significantly different (P < 0.001) with respect to the quality of the exhaust systems at workplaces for both the occupational groups (P < 0.05 in both areas). Use of waste storage receptacles at the workplaces was very rare, (P < 0.001) at both exposed sites. Moreover, poor ventilation status, (P < 0.001 for both exposed areas), and low sunlight penetrations into workplaces (P < 0.001 for both areas) were observed as compared to control areas. Fresh air circulation was poor at PNs sites (P < 0.001) and MCs workstations (P < 0.005). Tasks reportedly performed by MCs included; engine repair, oil changing, lubrication and basic maintenance of fuel systems etc., whereas the work tasks of the PNs included dent repair, abrading, cleaning, brushing, burning, cementing, chipping, fastening, repairing, sanding, and spray-painting. Apart from petroleum products, chemical composition of the materials used in a typical painting shop e.g. diluents, binder, thinner, hardener etc. contained various solvents comprised of aromatic hydrocarbons such as benzene, toluene and derivatives, whereas the main contaminants occurring in the mechanical workshops included kerosene oil, residues of petroleum products and used engine oil. Worker's clothing and exposed body parts were smeared with used engine oil to which they were constantly exposed overtime.

**Table 2:** Workplace hygiene status of exposed versus un-exposed workplaces

Workplace Description	Ranks	Painters group * (n = 20)	Mechanics group * (n = 20)	Control group * (n = 20)	**P-value **^**a**^	**P-value **^**b**^
**Mechanical Exhaust System**						
	Good	0 (0)	0 (0)	3 (15)	P < 0.027	P < 0.027
	Satisfactory	1 (5)	1 (5)	5 (25)		
	Poor	19 (95)	19 (95)	12 (10)		
**Workplace Ventilation**						
	Good	0 (0)	0 (0)	2 (10)	P < 0.001	P < 0.001
	Satisfactory	3 (15)	5 (25)	16 (80)		
	Poor	17 (85)	15 (75)	2 (10)		
**Sunlight Penetration**						
	Good	0 (0)	0 (0)	11 (55)	P < 0.001	P < 0.001
	Satisfactory	20 (100)	16 (80)	7 (35)		
	Poor	0 (0)	4 (20)	2 (10)		
**Workplace Overall Hygiene**						
	Good	0 (0)	0 (0)	16 (80)	P < 0.001	P < 0.001
	Satisfactory	1 (5)	3 (15)	4 (20)		
	Poor	19 (95)	17 (85)	0 (0)		

* Frequency (% of total)

** Chi-square P-values

^a ^Car-spray painters v/s Control

^b ^Mechanics v/s Control

The concentrations of NAPH in blood were described as median, skewness, minimum and maximum, for exposed (MCs and PNs) and unexposed (control) groups (Tables 3 &4). Based on skewness indicator range between -1 and 1, the data for blood NAPH level was non-normal or skewed. Comparison between MCs and PNs showed significantly higher blood NAPH concentrations in MCs as compared to PNs with median NAPH values of 333.0 and 131.8 μgL^-1 ^respectively. A significantly higher blood NAPH concentration in MCs as compared to the control group (P < 0.001) was observed while this difference was not statistically significant between PNs and control group.

**Table 3:** Blood naphthalene concentration (μgL^-^^1^) of occupationally exposed workers and un-exposed (control) group

Descriptive	Exposed			Un-Exposed
	
	Total (n = 60)	Painters (n = 20)	Mechanics (n = 20)	Control (n = 20)
Not detected (n/total)	28/60	6/20	7/20	15/20
Detected (n/total)	32/60	14/20	13/20	5/20
Median *	155.9	131.8 ^**P**^	333.0 ^**M**^	114.1 ^**C**^
Skewness	2.2	2.0	1.2	1.8
Minimum detected	56.0	54.1	53.7	56.0
Maximum detected	1980.6	892.9	1980.6	632.4

*Comparisons (Mann-Whitney U-test) of blood naphthalene levels for: P = Painters, M = Mechanics, C = Control. *P *= 0.168 for P vs C; *P *< 0.001 for M vs C; and *P *< 0.005 for M vs P.

**Table 4:** Smoker and non-smoker wise comparison for blood naphthalene concentrations (μgL^-1^) in occupationally exposed workers and un-exposed (control) group

Descriptive	Exposed				Un-Exposed	
	
	Car-Spray Painters (n = 20)		Car-Mechanics (n = 20)		Control (n = 20)	
Sub-groups	Smoker	Non-smoker	Smoker	Non-smoker	Smoker	Non-smoker
Not detected (n/total)	2/12	4/8	3/9	4/11	1/3	14/17
Detected (n/total)	10/12	4/8	6/9	7/11	2/3	3/17
Median	140.3	27.0	280.9	192.3	109.5	17.7
Skewness	84.7	25.4	135.5	117.2	98.5	13.6
Minimum detected	72.8	54.1	79.1	53.7	109.6	56.0
Maximum detected	892.9	92.4	1980.6	1618.9	632.4	249.1

SMs vs NSMs*	Significant at P < 0.05		Significant at P < 0.05		Significant at P < 0.001	

* Mann-Whitney U-test for comparison of median values between smokers and non-smokers

**Figure 1:**
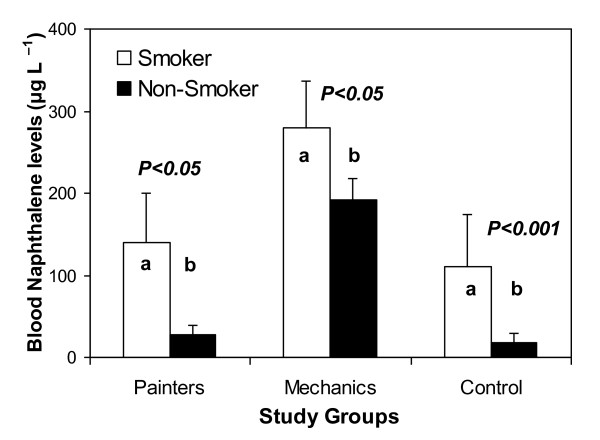
**Comparison of blood naphthalene concentrations between smokers and non-smokers in occupational and control groups. **Bars with different letters are significantly different.

**Figure 2:**
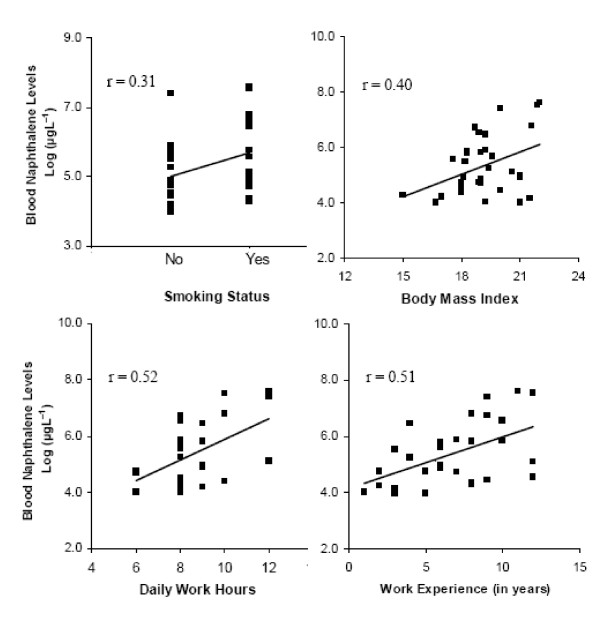
**Relationship between log-transformed values of blood naphthalene levels of exposed subjects (n = 32) with smoking status, BMI, daily work hours and work experience (years spent in job)**.

Split data for smokers (SMs) and non-smoker (NSMs) categories in each study group showed significantly higher NAPH levels among SMs than NSMs in both exposed groups (P < 0.05). Blood NAPH was also higher in SMs from control group as compared to NSM control subjects illustrated through Mann-Whitney comparison of median values (Table 4 and Figure 1). Log-transformed laboratory data for exposed workers (n = 32) showed a positive correlation between the blood contaminant level and variables of interest, such as smoking habit, total years spent in job (work experience), BMI and daily work hours (Figure 2). SMs were observed to have more NAPH in their blood, which was positively correlated smoking habit. Log-transformed data for blood NAPH concentration also showed a strong correlation with work hours and work experience.

## Discussion

In Pakistan, worker safety is largely neglected aspects and little information on contaminant exposure is available because surveillance studies have never been given priority. Our investigation therefore pioneers the monitoring of chemical exposure in selected occupations. In this effort, assessment of exposure to PAHs was carried out by measuring blood concentrations of NAPH. The survey primarily focused on workers in two occupations where there is routine exposure to PAHs, but also took into account several environmental parameters and the personal behaviours of the workers that may enhance their risk of exposure. One of the striking results from our investigation was the observation that MCs had significantly higher concentrations of NAPH in their blood serum than those that were measured for PNs and the control group. As in many other countries, automobile mechanical work is a common occupation in Pakistan. According to Bureau of Labour Statistics [15], in 2008, there were about 763,700 people working as automobile technicians and mechanics in the US. There are no similar census data for Pakistan, but there are large numbers of automobile workstations and car-spray painting shops in the urban areas of Rawalpindi and Islamabad. These occupations provide opportunities for earning wages at an early age, and the majority of these workers come from poor communities in which families cannot afford educational expenses and thus send their children to work as apprentices to support their family. Thus a great number of these workers are self-employed and have not acquired any proper technical education. Scarce use of self protective equipment by these workers further increases the threat of exposure to inhalable volatile chemicals both via alveolar and dermal routes of absorption.

Occupational hazards vary in relation to socio-economic factors, and our survey showed that exposed groups (MCs and PNs) had poor socio-economic backgrounds with low levels of education, and were not much aware of chemical hazards. Education is not only very important to improve work related expertise, but it is also necessary for a worker's own betterment, because health information enables one to cope with health related problems and it motivates and enhances the capabilities of individuals to solve personal health problems [16]. Income status of these subjects was also relatively low (i.e. median monthly income Rs. 8.5 thousand or $100 US) in both occupations. Although subjects in our study appeared to be physically healthy, their generally poor socio-economic status increases their likelihood of employment in occupations where they will be exposed to environmental contaminants. NAPH is a ubiquitous PAH, which is classified as a potential carcinogen, and as such is also a good marker of exposure to PAH at worksites. Moreover, as it can exist in gas phase, this chemical can be used for air and biological monitoring of exposure to PAHs [8,9]. According to Unwin NAPH dominates the PAH profile at every site, being a representative of 50-90 percent of total PAHs [3]. The United State's EPA has declared NAPH as among the most hazardous of air pollutants [17]. Therefore, the detection of higher blood levels of NAPH in our occupational groups suggested not only a considerable risk for their health but also its prevalence in the occupational environment in Pakistan.

Relatively higher blood NAPH levels were discovered among MCs, and were significantly elevated as compared to those measured for the control group (P < 0.001) and PNs (P < 0.005). The maximum blood NAPH concentration for this group was 1980.6 μgL^-1^, which is about 80 percent higher than previously reported in India [5], indicating that PAH exposure at mechanic workplaces was relatively higher as compared to other studied areas. It is likely that this higher level of exposure to PAHs among MCs is due to exposure to various petroleum products that MCs come in contact with during car-repair work and from the ambient environment. For example, used gasoline engine oil (UGEO) is a common source of PAHs, to which mechanics routinely come into direct contact. Although fresh oil also contains PAHs in small amounts [18] used oil and UGEO contains up to 70 percent PAHs, especially those having more than three rings [18,19]. Oil that is used for lubrication of engine crankcases contains many aromatic compounds, especially NAPH, phenantharene, fluorene [19] pyrene, benzanthracene, and benzo(a)pyrene [20]. These PAHs are extracted by lubricating oil and are mostly retained in engine crankcases [21]. PAH concentrations in the oil increase over time [21,22], such that long periods between oil changes increases the exposure risk of mechanics to PAHs [20]. Waste crankcase oil may also contain a higher percentage of other toxic aromatic compounds [18]. UGEO is thus known to be very toxic to humans. Our results provide strong evidence that blood NAPH levels are occupationally related, even after taking into account the effects of other factors like smoking. The results further suggest that MCs are more frequently exposed to PAHs at mechanic workstations than do workers at spray-painting sites. It appears that occupational exposure varies with the nature of job and workplace description, and that MCs face greater occupational health risks than the general population.

We also examined occupational hygiene conditions in our surveyed locations, which highlighted some important determinants of exposure in the workplace that have not been discussed much in the literature. Our study documented that overall hygiene and ventilation status were poor in the workplaces we surveyed (Table 2). Both MCs and PNs explicitly categorized their workplaces as poor due to the presence of spills, residues of used volatile and non-volatile chemicals. In the case of oil and PAHs which are mostly non volatile, skin absorption is probably the major route for exposure. Because human skin has direct contact with the environment, it serves as a major entry route for many chemicals. Proper ventilation systems on the other hand are necessary to minimize the air born burden of chemicals emerging from various onsite processes [23]. We found both poor ventilation and improper workplace hygienic conditions (Table 2), which may continuously be injurious to the health of workers.

Occupational exposure was quite evidently observed in our study upon comparing the NAPH levels among NSMs for both MCs and PNs. Apart from occupational exposure, our results showed that NAPH in SMs was 1.7 times higher than NSMs among mechanics (Table 4) and this difference was significant (Figure 1) which clearly indicated that smoking is an additional risk factor. Despite the selective response of our investigated groups towards smoking habit, a similar trend in blood NAPH concentration for PNs i.e. 6.5 times higher NAPH in blood of PNs for those who smoke than those who did not, further strengthens this finding. In addition to this, correlation between blood NAPH levels and passive smoking (i.e. SMs at home r = 0.59 and r = 0.62 among PNs and MCs respectively) showed that passive smoking may also be an important source of PAHs exposure. We observed an intriguing effect of cigarette smoke both for smokers and those who had been passively exposed to cigarette smoke, which is obvious from the split data of SMs and NSMs among the MCs, PNs and control groups (Table 4). This led us to believe that passive smoking is also a cause of exposure and it is equally threatening to population even if they are not active smoker. The split data for SMs and NSMs from the control group also showed that there was ten times higher blood NAPH in the SMs category, confirming that smoking creates a perilous environment for local people who do not have any direct occupational exposure to chemicals but they get exposures to PAHs in varying degree from either active or passive smoking [24]. Smoking is an important route of direct exposure to PAHs and a well known source of many other chemicals [25,26]. Normally tobacco smoke results in exposure to several carcinogenic PAHs such as B[a]P [27]. Although in most of the cases occupational exposure dominates in both SMs and NSMs, which had 2-20 folds greater NAPH concentrations than control subjects [28], our results indicated that smokers in this occupation receive additional exposure to PAHs from inhalation of tobacco smoke. The tendency for smoking addiction is likely also related to socio-demographic factors (e.g. age and income) in Pakistan [29].

We also draw inferences from the correlation analysis of our log-transformed laboratory data with work related parameters, i.e. daily work hours and work experience as well as BMI of the subjects (Figure 2). The body burden of PAHs which, being lipophilic in nature, are hypothesized to increase over time and in proportion to the BMI [30] as fatty tissues will represent a repository for PAHs [31]. BMI is known to affect circulating levels of lipophilic contaminants, which are stored in adipose tissue [30]. Exposure time is an important determinant of increased body burden of many contaminants. In the case of PAHs, a positive relation with exposure time has been documented in a prior study on the blood of children in Lakhnaow [32]. Risk of exposure among mechanical workers is likely to increase when they spend long hours dealing with crankcase used oil, especially in the absence of protective equipment and under the poor hygienic conditions [33,34]. Viegas et al [35] had considered a moderate positive correlation between duration of occupational exposure to formaldehyde in terms of years of exposure and frequency of micronuclei in peripheral blood lymphocytes. Our correlation result for work experience (years spent on the job) is in agreement with these findings and can be helpful in future studies to assess the health risk of exposed workers.

It is clear that occupational exposure to PAHs is influenced by multiple factors and that prevailing workplace conditions for MCs demands a good hygiene program and proper ventilation to minimize accumulation of chemicals indoors. At the same time, risk of PAH exposure is also associated with daily work hours. Smoking contributes and enhances exposure risks to PAHs, but at the same time work duration and job experience appeared to be more influential and were consistently related to the body burden of PAHs due to exposures at the workplace. Our results suggest that considerable improvements in workplace hygiene are needed to minimize health risks related to these occupations.

## Conclusions

In Pakistan, legislation to ensure worker safety is almost nonexistent and no guidelines have been provided for permissible safe exposure limits by the health authorities. Moreover, despite our best efforts, we could find no literature pertaining to occupational risk assessments for Pakistan. The two occupations we selected for this survey are representative of those performed by low income workers in Pakistan. Most of the subjects we studied were self employed or working autonomously under the supervision of senior mechanics and were randomly located in both commercial and residential areas. Our findings revealed that automobile-mechanical workshops are potential exposure sites for PAHs, receiving greater exposure as compared to those employed as automotive spray painters. Blood levels of NAPH were also directly associated with smoking. SMs in all three groups had higher blood concentrations of NAPH, but the MCs due to their workplace environment and the nature of their job showed considerable susceptibility to the combined effects of smoking and occupational exposure. Poor workplace hygiene and improper ventilation are among key factors accounting for potential risks. Blood NAPH levels were further correlated with daily work hours and work experience showed that spending long hours in a poor hygienic environment increased susceptibility to chemical exposures. Therefore, a continuous monitoring seems imperative not only to reduce exposure risks, but also for assessment of environmental hygiene conditions. There are many other workers such as those employed as petrol pump and highway toll bar attendants, bus and taxi cab drivers, who also have risks of PAHs exposure and thus should be included in future studies. Moreover, we highly emphasize the need for better record of occupational injuries and establishment of a better health monitoring system for workers in tasks where they are exposed to PAHs.

## List of Abbreviations

EPA: Environmental protection agency; HPLC: High performance liquid chromatography; LOD: Limit of detection; LOQ: Limit of quantification; MCs: Car-mechanics; NAPH: Naphthalene; μgL^-1^: Microgram per liter; NSMs: Non-smokers; PAHs: Polycyclic aromatic hydrocarbons; PNs: Car-spray painters; PPM: Parts per million; SMs: Smokers; UGEO: Used gasoline engine oil.

## Competing interests

The authors declare that they have no competing interests.

## Authors' contributions

The research idea was conceived and designed by AK and AR with some modifications suggested by MQ. The data was analyzed by AK and manuscript wrote collectively by AR, AK and IUC. All authors agreed upon the final shape of manuscript after reading.

## Pre-publication history

The pre-publication history for this paper can be accessed here:

http://www.biomedcentral.com/1471-2458/11/467/prepub
